# JOR Spine: Reaping the harvest of a community effort

**DOI:** 10.1002/jsp2.1288

**Published:** 2023-09-29

**Authors:** Robert L. Mauck, Mauro Alini, Daisuke Sakai

**Affiliations:** ^1^ University of Pennsylvania McKay Orthopaedic Research Laboratory, 114A Stemmler Hall, 36th Street and Hamilton Walk, Philadelphia Pennsylvania US; ^2^ AO Foundation, AO Research Institute Clavadelerstrasse 8, Davos, Platz Graubünden Switzerland; ^3^ Tokai University School of Medicine Graduate School of Medicine Orthopaedic Surgery, 143 Shimokasuya Isehara Japan

Dear readers,

As the seasons begin to change (at least in the northern hemisphere), we turn our attention to gathering the fruits of our collective labor and start our planning for the coming year. Since the groundwork and planning began in earnest in the fall of 2017, to our first issue in March of 2018, to the present day, the ‘seeds’ that were the JOR Spine concept have come to fruition, and we can now all share in the bounty that is our JOR Spine journal. Over the last six years, the international spine community has come together to support this new effort, and we have steadily progressed though the benchmarks that signify stability and success of a new journal. Last summer we were delighted to receive our first impact factor, a major milestone. This past July, we were even more delighted to find out that our impact factor had held steady, at a 3.7. While impact factor is only one metric, we are happy that all of your labors, and excellent submissions, have had this result, and firmly establish the JOR Spine in the top echelon of Spine related journals.

As we think to the future, we are excited by plans for the coming year. The December issue is slated to be a ‘special issue’, with a compendium of manuscripts from the 2022 ORS/PSRS meeting. We thank the organizers for that great meeting and for assembling an outstanding lineup of papers that are being finalized. Many of you also just finished a race to the finish line for your ORS 2024 abstract submissions. We are excited to see all of the new spine‐focused work presented at the meeting, and to see it submitted to the JOR Spine soon! We also look forward to celebrating with all of you our next **JOR Spine Early Career Awardee**. As you'll recall from past years, this award, developed in collaboration with the ORS Spine Section, is designed to recognize the ‘rising stars’ of our field and highlight their outstanding work published in the *JOR Spine*. For each of the past four years, awardees have been recognized at the ORS Annual Meeting, and we look forward to continuing this tradition in the coming year. Applications for this highly competitive award are due October 30th, 2023 and information on the award nomination process and eligibility criteria can be found at: https://onlinelibrary.wiley.com/page/journal/25721143/homepage/earlycareeraward. Please consider nominating your colleagues (or yourself) for this Award!

As we move into the fall, we wish you all a bountiful harvest, and look forward to your excellent submissions and suggestions, the seeds that will continue our progress in building JOR Spine into the preeminent spine journal for our community.

Rob, Mauro, and Dai.

